# Hypertension as a prominent manifestation secondary to renal artery lesions in pediatric Behcet’s disease

**DOI:** 10.1186/s12969-023-00932-6

**Published:** 2024-01-19

**Authors:** Xinning Wang, Zhixuan Zhou, Jianguo Li, Gaixiu Su, Xiaohui Li

**Affiliations:** 1 Department of Rheumatology and Immunology, Children’s Hospital Capital Institute of Pediatrics, Beijing, China; 2 Department of Rheumatology and Immunology, Children’s Hospital Capital Institute of Pediatrics, Chinese Academy of Medical Sciences & Peking Union Medical College, Beijing, China; 3 Department of Cardiovascular Medicine, Children’s Hospital Capital Institute of Pediatrics, Beijing, China; 4 Department of Cardiovascular Medicine, Children’s Hospital, Capital Institute of Pediatrics, Chinese Academy of Medical Sciences & Peking Union Medical College, 2 Yabao Road, Chaoyang District, Beijing, China

**Keywords:** Behcet’s Disease, Children, Hypertension, Renal artery

## Abstract

**Objective:**

Hypertension caused by vascular Behcet’s disease (BD) is an important prognostic factor of paediatric BD. However, much less is known about its clinical features. The objective of this study was to investigate the clinical characteristics of paediatric vascular BD complicated by hypertension.

**Methods:**

A retrospective study was carried out in paediatric BD patients complicated by hypertension treated in the Children’s Hospital Capital Institute of Paediatrics from Jan 2009 to Dec 2022.

**Results:**

Of 65 BD patients, 6 (9.2%) were complicated by hypertension, 5 patients were female, and the median ages of onset and diagnosis were 9.8 years and 11.3 years, respectively. Three patients were found to have cardiac involvement and hypertensive retinopathy secondary to hypertension. Five of the 6 patients with hypertension had right renal artery involvement, and all of them were treated with glucocorticoids and immunosuppressants. Four patients were treated with biological agents. One patient with severe renal artery stenosis underwent unsuccessful vascular interventional therapy. After 3–6 years of follow-up, five patients were found to have renal atrophy, and one patient was at stable condition.

**Conclusion:**

Hypertension in paediatric BD is mainly caused by renal artery involvement. Early recognition and treatment of vascular involvement in BD is important to prevent poor prognosis.

## Introduction

Behcet’s disease (BD) is a chronic, recurrent systemic vasculitis syndrome with high heterogeneity. The most common manifestations include recurrent oral aphthous ulcers, genital ulcers, uveitis and cutaneous lesions [1]. Recently, more attention has been given to arterial involvement, although veins are predominantly involved [2]. In the past, attention was focused on venous involvement in Behcet’s disease. Some patients may have hypertension caused by renal artery involvement as a manifestation, which is difficult to notice. To date, few studies have reported renal artery involvement in adults [3]. Hypertension is an uncommon manifestation of BD but may cause severe complications that lead to poor prognosis or even death. However, there are limited reports on hypertension in paediatric BD. We conducted this study to investigate the prominent clinical characteristics of BD patients with hypertension aiming to make early diagnoses and treatments. The main clinical characteristics, treatment and long-term follow-up of paediatric BD complicated by hypertension were reported.

## Methods

This retrospective study was conducted at the Children’s Hospital Capital Institute of Paediatrics (CIP). Children diagnosed with BD from Jan 2009 to Dec 2022 were included in this study. All participants fulfilled the paediatric criteria for BD (PEDBD) (2015) [4]. Patients diagnosed with hypertension in this study fulfilled the revised criteria of the Clinical Practice Guidelines of the American Academy of Paediatrics [5]. The diagnosis of vascular artery involvement was based on clinical assessments and imaging data: Doppler sonography and/or angio-computed tomography arteriography (CTA) and angio-magnetic-resonance imaging (MRI) examinations. For peripheral blood vessels, the ultrasound Doppler method was used, and for axial vessels such as the thoracic aorta, CTA or MRI were used for detection. Clinical data, including age, sex, oral and genital ulcerations, blood pressure, cutaneous lesions, ocular lesions, arthritis, vascular lesions, neurological involvement, inflammatory laboratory parameters, treatment, and follow-up, were collected and analysed. This study was approved by ethics committees from the Capital Institute of Paediatrics (SHERLL2021047).

## Results

In a total of 65 paediatric BD patients in this study, six patients accounted for 9.2% of the BD patients and were complicated by hypertension. The main characteristics of the 6 patients are summarized in Table 1. Five of the 6 patients were female. The median age at diagnosis was 9 years (range: 4–15 years).

**Table 1:** Clinical manifestations of 6 BD patients with hypertension

PN(patient number)	Age(years)	Sex	Fever	PL/EN(papulopustular lesions/erythema nodosum)	Hypertension/Mean blood pressure	GU(genital ulcers[mmHg])	OAU(OAU)	NI(neurologic involvement)	Uveitis	Hypertensive retinopathy	ITI(intestinal tract involvement)	Cardiac involvement/ Echocardiographic findings•	Arthritis
1	8	F	Y	PL	Y(145/102)	N	Y	Y	N	N	Y	N	N
2	7	F	Y	N	Y(130/75)	Y	Y	N	Y	N	N	Y/Ejection fraction decreased	Y
3	12	F	Y	EN	Y(135/89)	Y	Y	Y	N	Y	Y	Y/left ventricle hypertrophy	N
4	13	F	N	PL	Y(135/90)	N	Y	N	N	N	ND	N	Y
5	15	M	N	PL and EN	Y(143/96)	Y	Y	N	N	Y	N	N	Y
6	15	F	N	N	Y(162/90)	N	Y	N	N	Y	Y	Y/ Left ventricular and aortic valve hypertrophy•	N

EL: eye lesions; EN: erythema nodosum; GU: genital ulcers; ITI: intestinal tract involvement; N: no; NI: neurologic involvement; ND: no data; OAU: oral aphthous ulcers; PN: patient number; PL: papulopustular lesions; Y: yes

### Vascular involvement

In the current study, patients accounting for 23.1% (15/65) were found to have vascular involvement. The vascular lesions in this study included stenosis, dilatation, wall thickening and Thrombosis. A total of 10.7% (7/65) of cases were found to have venous lesions, and 18.4% (12/65) were found to have artery lesions. Six patients had both arterial and venous involvement, and eight patients had only arterial involvement. Five cases were complicated with Thrombosis. Two patients were found to have intracranial venous sinus Thrombosis and pulmonary artery Thrombosis.

Of the 12 patients complicated with artery lesions, 8 had renal artery involvement, and 6 presented with hypertension. Six of the 8 patients had involvement of the right renal artery. Six patients were found to have renal artery involvement at the same time as the BD diagnosis, while in the remaining two patients, renal artery lesions were found later after the diagnosis of BD. There were 6 cases of unilateral renal artery involvement and 2 cases of bilateral involvement.

### Hypertension

Five of the 6 patients complicated with hypertension in this cohort suffered from grade 2 hypertension, and 1 patient had grade 1 hypertension. The average blood pressure is shown in Table 1. Two patients developed hypertensive encephalopathy during the course of the disease, and the blood pressure of the 2 patients was over 180/120 mmHg when encephalopathy occurred. In 5 patients, hypertension was found through a physical examination during the first hospitalization. In 1 patient, the blood pressure was normal in the first physical examination (unilateral limb), but hypertension was found when hypertensive encephalopathy occurred. Cardiac involvement and hypertensive retinopathy secondary to hypertension were found in 3 patients.

### Involvement of extravascular organ systems

Oral aphthous ulceration was observed in 6 patients in this cohort. Other manifestations included cutaneous lesions (nodular erythema, purulent herpes), enteral nervous system involvement and arthritis in 5 patients, fever in 4 patients, and gastrointestinal tract lesions in 3 patients. The pathergy reaction was negative in 6 patients. The renal function of 6 patients was normal, and 1 patient had protein in the urine. Compared with patients with nonvasculo-BD, there was no significant difference in the manifestations.

### Laboratory investigations

The leukocyte and platelet counts were increased in all 6 patients with hypertension, and haemoglobin was decreased in 5 patients. The inflammatory indexes, including erythrocyte sedimentation rate (ESR) and C-reactive protein (CRP), were significantly elevated in 6 patients. The average CRP and ESR results were 92.9 mm/h and 100.2 mg/dl, respectively. Two patients were positive for anti-nuclear antibody (ANA), and two were positive for anti-neutrophil cytoplasmic antibody (ANCA) (Table 2). The activity of the renin angiotensin aldosterone system was significantly increased in 4 patients who were tested. The pathogenic examination was positive in all 6 patients, including 2 cases of mycoplasma infection, 1 case of Streptococcus infection, and 1 case of parvovirus B19 infection.

**Table 2:** Laboratory examination of 6 BD patients complicated with hypertension

Laboratory examination	Results
Leukocyte ↑	5/6(83.3%)
Anemia	5/6(83.3%)
Platelet ↑	6/6(100%)
Ferritin ↑	5/6(83.3%)
ESR ↑	6/6(100%)
CRP ↑	6/6(100%)
Cytokines	
---TNF-α↑	6/6(100%)
---IL-6↑	6/6(100%)
---IL-2R↑	6/6(100%)
ANA+	2/6(33.3%)
ANCA+	2/6(33.3%)

### Treatment and follow-up

All patients received treatment with glucocorticoids and immunosuppressants (Table 3). As the most commonly used immunosuppressant, cyclophosphamide (CTX) was used in 5 patients. Other immunosuppressants included thalidomide (*n* = 2), methotrexate (MTX) (*n* = 2), and mycophenolate mofetil (MMF) (*n* = 2). Three patients (Patients 1, 5, and 6) were treated with tumour necrosis factor-α.

**Table 3:** Blood pressure and treatment in children with BD with renal artery involvement

Patient Number	Blood pressure(L/R)	RAS activation	Renal artery stenosis	Renal atrophy	Abnormal renal function (urinalysis, serum creatinine)	Markers of renal injury elevation	Glucocorticoid	Anti-hypertension drugs
1	L 146/116 R 144/106	Y	Y	Y	Y	Y	MPPT(10 mg/kg.d*3days), prednisone oral(1 mg/kg)	Amlodipine(0.23 mg/kg.d) prazosin hydrochloride(0.05 mg/kg.d)
2	L 136/52 R 133/50	ND	Y	Y	N	ND	MPPT(10 mg/kg.d*3days twice), prednisone oral(1 mg/kg.d)	Amlodipine(0.34 mg /kg.d)
3	L 128/102 R 140/108	Y	Y	Y	N	Y	prednisone oral(1.4 mg/kg.d)	Amlodipine(0.33 mg /kg.d) Captopril(1.27 mg/kg.d) Metoprolol(1.69 mg/kg.d)
4	L 125/70 R 135/80	ND	Y	N	N	Y	MPPT(10 mg/kg.d*3days ) prednisone oral(1 mg/kg.d)	Amlodipine(0.30 mg /kg.d) Captopril(1.02 mg/kg.d)
5	L 138/94 R148/98	Y	Y	Y	N	Y	MPPT(10 mg/kg.d*3daystwice), prednisone oral(1 mg/kg.d)	Nifedipine nifedipine sustained-release tablet(0.6 mg/kg.d) losartan potassium(1 mg/kg.d) spironolactone(0.4 mg/kg.d) Prazosin (0.08 mg/kg.d)
6	L 149/97 R 154/77	Y	Y	Y	Y	Y	MPPT(20 mg/kg.d*3days*4 times), prednisone oral (0.8 mg/kg.d)	Spironolactone(0.67 mg/kg.d) Metoprolol(0.76 mg/kg.d) Prazosin(0.05 mg/kg.d)

(TNF-α) inhibitors. Five patients were treated with anticoagulant or antiplatelet medications with warfarin or low-dose aspirin. No haemorrhagic complications were observed in a patient under anticoagulant or antiaggregant therapy. Six patients were followed up for 3 years to 6.4 years. Patients 1 and 6 were found to have renal atrophy at the time of diagnosis. For patient 1, during the follow-up, the size of the right kidney progressively decreased 3 years after the first CT scan (Fig. 1A and B). She underwent balloon dilatation of the right renal artery but failed due to severe stenosis of the renal artery (Fig. 1). 1 C). Patient 4 relapsed because she stopped treatment without authorization. She had hypertension caused by stenosis of the renal artery and renal atrophy, and an incomplete renal cortex was found 2.5 years after the diagnosis (Fig. 2A and B). However, there was no corresponding clinical manifestation except the increase in blood pressure until she manifested with sudden convulsions caused by hypertensive encephalopathy.

**Fig. 1:**
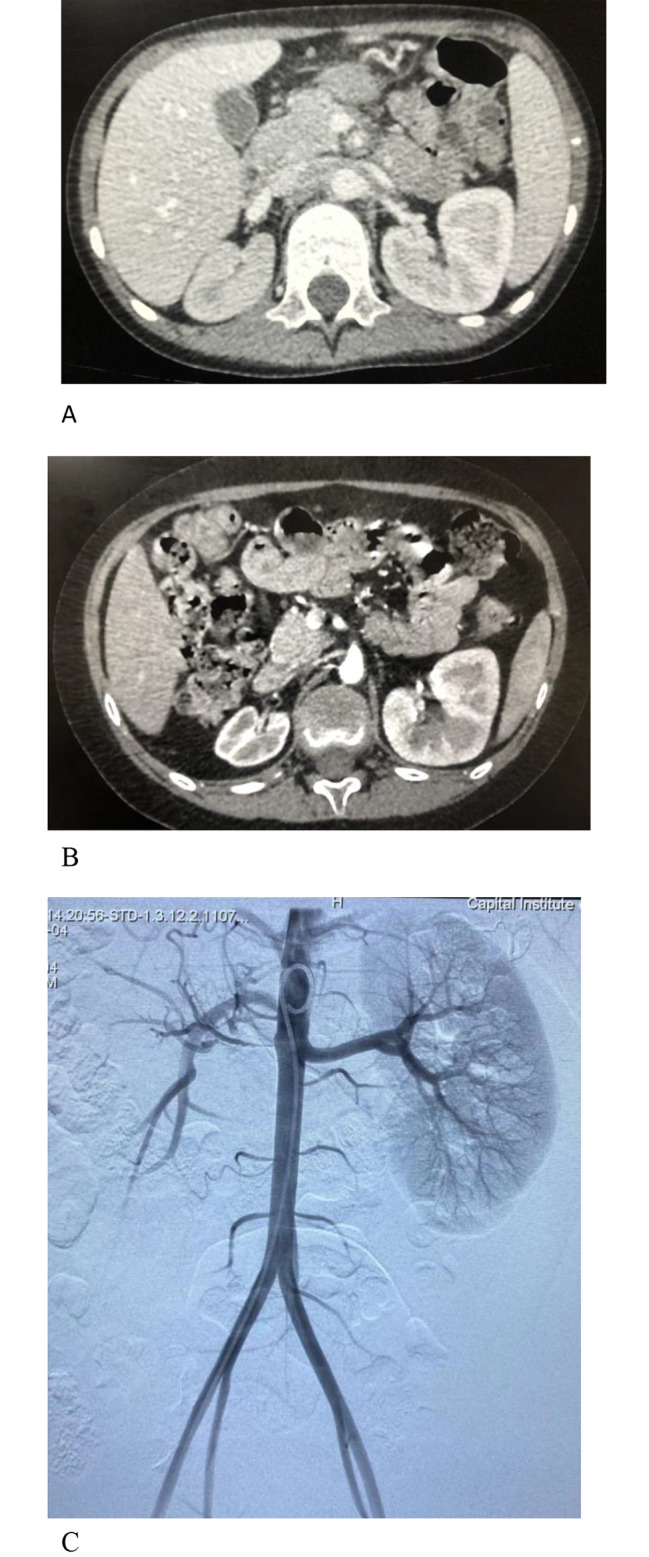
Patient 1, an 8-year-old girl, axial contrast-enhanced CT imaging. **A** showeds the narrowing of the right renal artery (arrow). Note the accompanying small renal size and deficient enhancement in the renal parenchyma. **B** 3 Three years later, a follow-up CT image demonstrated that the right kidney became further smaller had decreased in size. **C** This is an intraoperative imaging of the child, and the right renal artery was almost occluded so that there was very little blood supply to the right kidney, and the interventional guide wire was unable to pass through the stenosis

**Fig. 2:**
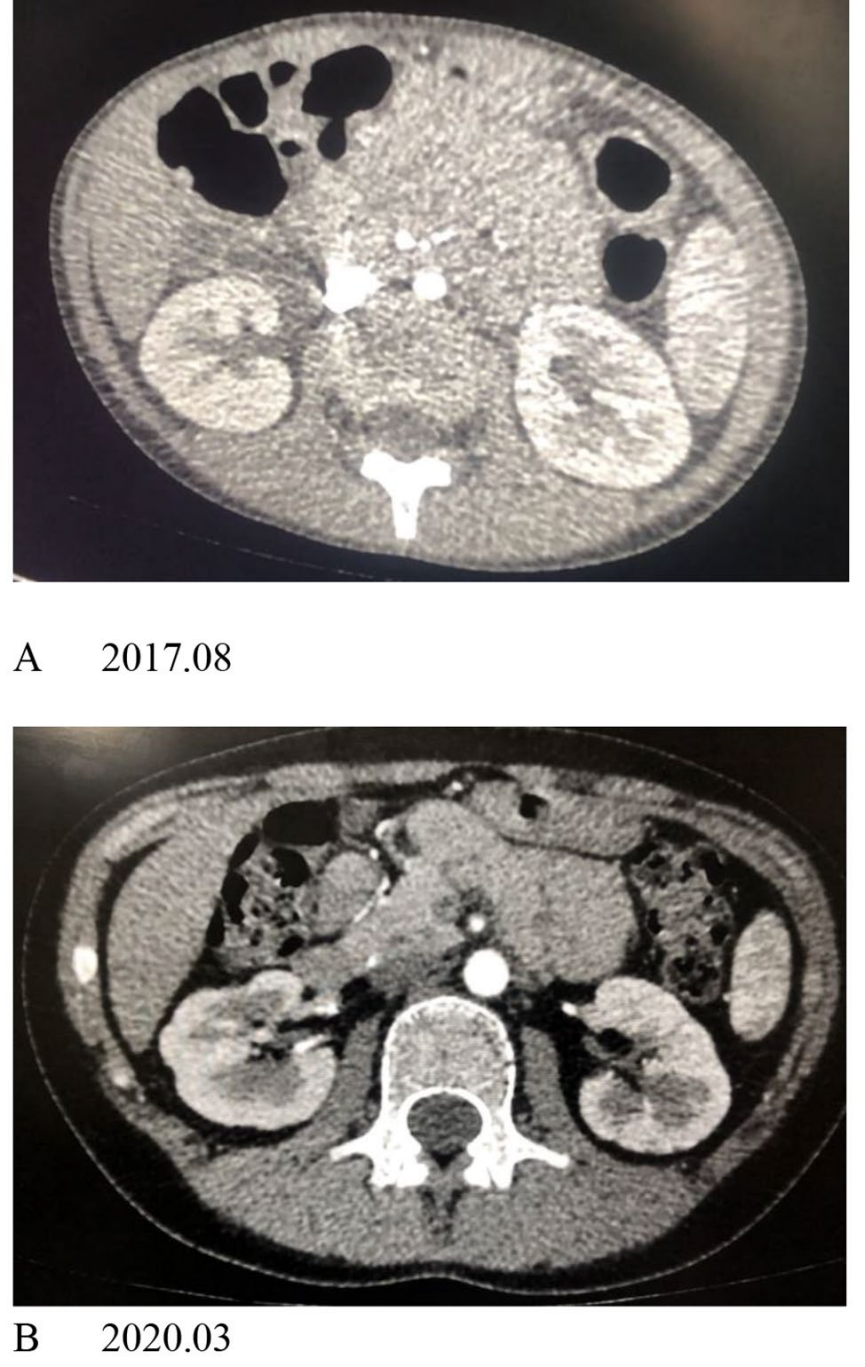
Patient 3, a 12-year-old girl, underwent axial contrast-enhanced CT imaging. **A** showeds accessory renal artery stenosis of the right kidney; the renal volume was normal with a smooth margin. **B** After 31 months, the edge of the right kidney became irregular due to focal thinner renal cortices

## Discussion

BD is a multisystem vasculitis syndrome that can involve blood vessels of all types and sizes and affect any tissue or organ [6]. Sixty-five BD patients were analysed, and 6 patients manifested hypertension in the current study. The common feature of these patients is renal artery stenosis as the cause of hypertension. This study expanded the spectrum of childhood Behcet’s disease and revealed important prognostic factors.

Vascular BD has been adopted for patients in whom vascular manifestations are present, and vascular manifestations often dominate the clinical features. Vascular lesions, with an incidence of 12.8%, are one of the main clinical manifestations of BD and may represent a life-threatening condition [7, 8]. Male sex and young age were reported to increase the risk of vascular complications. Vascular lesions occur in 1.8–32.1% of paediatric patients [9]. In China, the incidence of vascular lesions in adult BD is 7.7% [10]. In the past, venous involvement was frequently reported in many studies, while reports about arterial lesions in patients with BD are rare and often lack awareness. It was reported that the frequency of arterial lesions ranges from 1 to 33.5% [11–13]. In the current study, the frequency was 18.5% (12/65). All 6 patients with hypertension suffered from grade 2 hypertension and hypokalaemia caused by the increased production of renin due to stenosis of the renal arteries. For patients with hypertension caused by renal artery involvement, hypertension is a progressive process. During the early stage of artery wall thickening, blood pressure could be normal. With the progression of the disease, lumen stenosis may develop and lead to renin-angiotensin-aldosterone system activation, resulting in hypertension.

The patient could be asymptomatic, and the urine test and serum creatinine levels could be normal in the early stage of disease. Without a careful and systemic physical examination, it is difficult to detect hypertension and vascular involvement. It will be more difficult and confusing, especially in situations in which the blood pressure is normal at the beginning, which easily leads to ignorance of regular monitoring of blood pressure. In fact, the blood pressure of some patients was normal at the first hospitalization but monitoring after discharge was ignored, such as what happened with patient 1 and patient 3 in this cohort. The blood pressure of the two patients was normal at the beginning and was not monitored regularlyduring disease until they developed hypertensive encephalopathy years later, and the patients were screened for vascular involvement due to the hypertension. What should be noted and can be easy to ignore is regular bilateral blood pressure monitoring. There may be two reasons for this: on the one hand, the blood pressure was indeed normal at the beginning, and the other reason may be that the patient’s bilateral blood pressure was asymmetric, with the normal blood pressure value being obtained by measuring the blood pressure on the normal side. Therefore, it is important to measure the bilateral blood pressure routinely for vascular BD patients. Vascular ultrasound or CTA could help determine whether vascular involvement is present. Once vascular lesions occur, blood pressure should be closely monitored, and hypertension should be treated in a timely manner to prevent damage to important target organs, such as the eyes, heart, brain and kidneys. This finding reminds us that systematic screening for asymptomatic arterial lesions should be performed in BD rather than venous lesions only to find artery involvement in the early stage and treat in time to avoid important organ involvement. A careful physical examination is crucial in discovering hypertension and vascular murmur, which indicates vascular involvement.

Eight patients in this study had renal artery lesions, and five of them were found to have renal atrophy. Pyknosis related to hypertension and reduced renal blood supply may contribute to this phenomenon. What needs special attention is that five of the six patients had right artery involvement, and the pathogenic association between BD and right renal artery involvement needs to be clarified in future studies. It has been reported that in patients with fibromuscular dysplia (FMD), which often causes right artery stenosis, the right renal artery is more prone to be involved than the left renal artery, which may be due to renal mobility when assuming an upright position being greater in the right than in the left kidney [14]; therefore, it has been suggested that repeated stretching of the renal artery may cause microtraumas that predispose patients to FMD [15]. Therefore, we speculate that the susceptibility of BD patients to right renal artery stenosis may also be related to this factor.

Regarding vascular involvement, the prevalence in this study was 18.5% (12/65), of which renal artery involvement accounted for 12.3% (8/65). Other involved vessels include the main arteries and veins of the intracranial tract, limbs and organs, with a wide range of lesions. Previous studies have found that veins are more prone to be involved in BD and that vascular involvement, especially thrombosis, is common in men. In this study, the proportions of patients with arterial and venous involvement were similar, and most of the patients were female, which is different from previous studies. A study involving 796 Chinese patients with Behçet’s syndrome reported that 12.8% of the patients were affected in their blood vessels; the male/female ratio was 4:1; and the average age of onset was 29.5. In addition, 54.9% of patients with BD had arterial involvement, 70.6% of patients had venous lesions, and 25.5% had both arterial and venous involvement [8, 9]. This may be related to ethnic differences and the small number of patients within the cohorts. In summary,not only venous involvement, but also arterial involvement and thrombosis, should be given attention to in children with BD. Patients 2 and 4 were found to have multiple venous thrombi.

According to the laboratory examinations, leukocytes were increased slightly in 5 patients, and elevated platelets and mild anaemia were found in 6 patients. Notably, the inflammatory indexes (CRP and ESR) were increased in all patients; the average ESR was 90 mm/h; and the average level of CRP was 98.06 mg/dl. It is suggested that when the inflammatory index is significantly increased, attention should be given to vascular involvement. The pathergy reaction was negative in all patients. This might be related to the application of glucocorticoid therapy before the diagnosis. In this study, 5 patients were positive for an aetiology, suggesting that infection may play a role in the disease.

All children in this study were treated with glucocorticoids combined with cyclophosphamide, and other immunosuppressants included thalidomide and methotrexate. Three patients with nervous system involvement were treated with methylprednisolone pulse therapy, and two of them were treated with infliximab. Except for one patient who was lost to follow-up, the other patients were followed up for 3.3 years to 6.3 years. The symptoms and laboratory examinations improved, and the average time of clinical remission were 6 months. All three patients ad complications of severe renal artery stenosis. One patient stopped treatment one year after diagnosis and showed manifestations of hypertension and convulsions. Two years later, her kidneys had significantly shrunk, the renal cortices had became thinner, and parts of the renal cortices were discontinuous (Fig. 2A and B). This may be related to long-term hypertension and insufficient blood supply to the kidney. Three patients with venous thrombosis were treated with glucocorticoid pulses combined with TNF-α inhibitors, and one patient was treated with IL-6 inhibitors. Two of the patients were treated with steroid pulse therapy combined with cyclophosphamide. It is concluded that thrombosis in BD is mainly mediated by inflammation and that immunosuppressive therapy should be the core of treatment, which includes immunosuppressive drugs [16–18]. Anticoagulant therapy may significantly increase the risk of aneurysm rupture and fatal haemorrhage, and immunosuppressive therapy is important for patients with thrombosis [19, 20]. Hence, patients with venous thrombosis should undergo relevant examinations to detect the existence of pulmonary artery aneurysms.

A study showed that tumour necrosis factor inhibitors associated with other immunosuppressive drugs seem to be effective in the management of major vessel involvement and could reduce the risk of relapse [21, 22]. In the current study, αan ideal therapeutic effect was made with anti-TNF-α agents achieved for BD accompanied by pulmonary artery aneurysm and venous thrombosis, and the long-term prognosis should be discussed in further studies. For the treatment of hypertension, it is difficult to achieve the ideal effect. Usually, more than two kinds of antihypertensive drugs are needed (Table 3). Once renal artery stenosis occurs, it may lead to the activation of the renin-angiotensin system, and blood pressure is more difficult to control. The blood pressure of children with renal artery involvement but limited to the thickening of the wall was normal. Timely and effective treatment may prevent renal artery stenosis to protect important target organs. Therefore, routine examination of renal artery ultrasound or CTA in children with BD is as important as monitoring blood pressure.

The limitation of the current research is the small number of patients due to the low incidence. In the long run, multi-centre research is needed to identify genes that predispose patients to the condition and improve the detection and quantification of renal artery stenosis.

## Conclusions

In conclusion, hypertension in children with BD is mainly caused by renal artery stenosis, and early diagnosis is difficult. Renal artery involvement in BD in children is a serious complication and an important factor determining the prognosis of the disease. A long course of disease and lack of treatment may lead to renal atrophy. Patients with renal artery involvement should be closely monitored for blood pressure and regularly evaluated for important target organs. Early recognition of vascular involvement is important to evaluate prognosis.

## Data Availability

All data generated or analysed during this study are included in this published article.

## Abbreviations

ANA: Anti-nuclear antibody
ANCA: Anti-neutrophil cytoplasmic antibody
BD: Behcet’s disease
CRP: C-reactive protein
CT: Computed tomography
CTX: Cyclophosphamide
EL: Eye lesions
EN: Erythema nodosum
ESR: Erythrocyte sedimentation rate
FMD: Fibromuscular dysplia
GU: Genital ulcers
ITI: Intestinal tract involvement
MMF: Mycophenolate mofetil
MTX: Methotrexate
N: No
NI: Neurologic involvement
ND: No data
OAU: Oral aphthous ulcers
PN: Patient number
PL: Papulopustular lesions
PR: Pattern reaction
TNF-α: Tumor necrosis factor-α
Y: Yes
